# Risk Factors for Sensorineural Hearing Loss in Children and Adolescents with Sickle Cell Disease

**DOI:** 10.22038/ijorl.2025.74314.3500

**Published:** 2025

**Authors:** Amos Solomon, Nurudeen Adebola Shofoluwe, Hafsat Ahmad, Abdulkadir Isa, Shuiabu Iliyasu Yunusa, Hamza Anka Manir

**Affiliations:** 1 *Department of Pediatrics, Faculty of Clinical Scinces, College of Medical Sciences, Ahmadu Bello University and Ahmadu Bello University Teaching Hospital, Zaria, Kaduna State, Nigeria.*; 2 *Division of Otorhinolaryngology, Department of Surgery, Faculty of Clinical Scinces, College of Medical Sciences, Ahmadu Bello University and Ahmadu Bello University Teaching Hospital, Zaria, Kaduna State, Nigeria.*; 3 *Department of Otorhinolaryngology, Aminu Kano Teaching Hospital, Kano, Nigeria.*

**Keywords:** Risk factors, Sensorineural hearing loss, Sickle cell disease, Steady state

## Abstract

**Introduction:**

Sickle cell disease (SCD) is a major global health burden with significant clinical, social, and economic impacts. Sensorineural hearing loss (SNHL) is an underreported complication of SCD that is, primarily attributed to vaso-occlusive crises and ischemia. This condition adversely affects the quality of life, education, and social integration, particularly among children in resource-limited settings. Understanding the risk factors for SNHL is crucial for prevention, early detection, and timely intervention. This study evaluated the prevalence of SNHL in children with SCD and identified associated risk factors.

**Materials and Methods:**

This prospective comparative study was conducted at a tertiary healthcare facility in Northwest Nigeria. A total of 250 children aged 5–16 years were enrolled, comprising 125 patients with confirmed sickle cell disease (SCD) in a steady state and 125 age- and sex-matched controls with a normal haemoglobin genotype (HbAA).

**Results:**

Bilateral SNHL was identified in 25.6% of SCD cases, whereas no SNHL was observed in the control group. The male-to-female ratio among the affected children was 1.2:1. Multivariate logistic regression revealed significant associations between SNHL and elevated white blood cell count (Odds Ratio {OR} 1.035; 95% Confidence Interval {CI} 1.020–1.050), elevated platelet count (OR 1.209; 95% CI 1.070–1.365), poor clinic attendance (OR 28.668; 95% CI 4.879–168.458; *P*= < 0.001), non-compliance with SCD medications (OR 9.634; 95% CI 1.830–50.718; *P* = 0.008), and frequent severe sickle cell crises requiring hospitalization (OR 2.106,; 95% CI 0.019–0.598; *P *= 0.001).

**Conclusion:**

This study highlights the high prevalence of SNHL in children with SCD and its association with modifiable risk factors. Routine audiological screening, consistent clinic attendance, medication adherence, and regular monitoring of haematological parameters are essential for early identification and management of SNHL. Targeted interventions can significantly improve the outcomes and reduce the burden of this debilitating complication.

## Introduction

Sickle cell disease (SCD) is an autosomal recessive hemolytic disorder characterized by the substitution of valine for glutamic acid at position six of the beta-globin chain, resulting in abnormal hemoglobin (HbS). This mutation leads to red blood cell deformity, increased fragility, and vaso-occlusion, contributing to a wide spectrum of SCD complications, including sensorineural hearing loss (SNHL) (1–3). SNHL is a well-recognized complication of SCD, with prevalence rates ranging from 3.8% to 33.5% among children and adolescents, which is significantly higher than those in the general population (4–15). Hearing loss ranges from mild to severe, and often progresses with age. Unfortunately, SNHL in SCD frequently develops insidiously and often presents with subtle or no symptoms until hearing loss becomes severe or irreversible (2,4–15). The underlying pathophysiology of SNHL in SCD involves microvascular occlusion during vaso-occlusive crises, which compromise oxygen delivery to the cochlea via the labyrinthine artery. This terminal artery has limited collateral circulation, rendering the cochlea particularly vulnerable to ischemia. Repeated sickling episodes result in blood stagnation, hypoxia, and ischemic damage to the cochlear hair cells and organ of Corti, culminating in progressive hearing loss. Although bilateral SNHL is the most common, unilateral cochlear hearing loss has also been reported (16–21).

Several risk factors have been linked to the development of SNHL in SCD, including frequent crises, early onset of crises, crisis severity requiring hospitalization, frequent blood transfusions, lack of specialist care, poverty, malnutrition, and abnormal hematological parameters, particularly elevated white blood cell and platelet counts (3, 22–24). This study aimed to evaluate the risk factors for SNHL among children and adolescents with SCD in a resource-limited setting in Northwest Nigeria. By embedding a case-control analysis within the SCD cohort, this study identified the clinical, laboratory, and sociodemographic predictors of SNHL. Unlike similar studies, this study also compared the prevalence of SNHL in SCD patients with that in age- and sex-matched controls with normal hemoglobin (HbAA). The findings from this study will enhance our understanding of the burden of SNHL in SCD and inform strategies for early identification, intervention, and prevention in affected populations.

## Materials and Methods

### Study Design and Setting

This prospective comparative study evaluated risk factors for sensorineural hearing loss (SNHL) in children with sickle cell disease (SCD). An embedded case-control study was conducted within the SCD cohort, comparing subgroups with SNHL (cases) and without SNHL (controls) to identify the clinical, laboratory, and sociodemographic predictors of SNHL. Additionally, a comparative analysis was performed between SCD cases and age- and sex-matched controls with a normal hemoglobin genotype (HbAA) to determine the prevalence of SNHL. The study was conducted weekly at a tertiary healthcare facility in Zaria, Northwest Nigeria, which houses a dedicated Sickle Cell Clinic serving 60–70 pediatric patients weekly.

### Study Population and Source

The study population included children and adolescents aged 5–16 years. Participants were recruited from two groups: children with sickle cell disease (SCD; cases) attending the Sickle Cell clinic and children with a normal hemoglobin genotype (HbAA; controls) attending the General Outpatient Department. All participants were enrolled during the study period after obtaining informed consent and assent.

### Inclusion Criteria:

SCD cases: Eligible participants had documented hemoglobin genotypes SS, SC, or SS+F and attended regular clinical follow-ups.

Controls: Children with confirmed HbAA genotypes.

### Exclusion Criteria:

SCD patients in acute crisis. Individuals with other hemoglobinopathies (e.g., HbAS, HbAC). History of hearing loss, ear surgery, head trauma, radiotherapy, stroke, chronic transfusions, excessive noise exposure, diabetes, HIV, tuberculosis, meningitis, mumps, measles, or ototoxic drug use.

### Sample Size Estimation

The required sample size was calculated using the single population proportion formula (25), with the following assumptions: 95% confidence interval, 5% margin of error, and 8% prevalence of elevated hearing thresholds based on prior studies in Southeastern Nigeria (17). This calculation yielded the required sample size of 113. To account for a 10% non-response rate, the final sample size was increased to 125 SCD patients and 125 age- and sex-matched controls with normal hemoglobin (HbAA).

### Sampling Technique

Systematic random sampling was employed. Participants meeting the inclusion criteria were randomly selected using a random number table generated based on the total number of attendees at the weekly SCD clinic (cases) and General Outpatient Department (controls). Recruitment occurred over 24 months (January–December 2021), with an average of 5 participants (cases and controls) enrolled weekly. All SCD participants underwent pure-tone audiometry (PTA) and tympanometry on the same day, while the hemoglobin genotype of controls was confirmed via electrophoresis prior to audiological assessments.

### Data Collection

Data were collected using a pretested, structured interviewer-administered questionnaire designed to capture sociodemographic characteristics, clinical history, laboratory results, and audiological findings. Sociodemographic and clinical data were obtained through face-to-face interviews with participants or their caregivers, including retrospective recall of SCD-related events (e.g., crisis frequency, age at diagnosis, and hospital- ization history). Laboratory data, including hematological parameters, were extracted from medical records during steady-state periods.

### Diagnosis of SCD and HbAA Confirmation

SCD diagnoses (HbSS, HbSC, or HbSS+F) were verified from the medical records. The HbAA status of the controls was confirmed using hemoglobin electrophoresis during the study.

### Venous Blood Collection

Venous blood samples (3–5 mL) were collected under aseptic conditions from all participants in EDTA-anticoagulated tubes. For SCD cases, samples were used for the full blood count (FBC) and differential tests. Controls underwent FBC, differential testing, and hemoglobin electrophoresis. The samples were stored and transported at 2–8°C, avoiding hemolysis, and analyzed within 6 hr to ensure accuracy.

### Audiological Assessments

Audiological evaluations began with a physical examination by an otolaryngologist to exclude middle ear pathologies (e.g., infections, wax impaction, and tympanic membrane perforations). Participants with impacted earwax were removed, and testing was postponed for one week. Middle ear function was assessed by tympanometry using a TYMP 87 Clinical Middle Ear Analyzer (GN Otometrics, Copenhagen, Denmark). Participants with normal type A tympanograms and intact acoustic reflexes underwent pure-tone audiometry. Diagnostic audiometry was performed in a soundproof double-walled booth using a calibrated Madsen Itera audiometer (GN Otometrics) with TDH-35 earphones. Air conduction thresholds were measured at 125 Hz, 250 Hz, 500 Hz, 1 kHz, 2 kHz, 4 kHz, 6 kHz, and 8 kHz. Hearing loss was classified according to the World Health Organization (WHO) criteria. Individuals with hearing thresholds between 10 dB and 25 decibels (dB) HL were categorized as having normal hearing. Those with thresholds ranging from 26 to 40 dB HL were classified as having mild hearing loss. Hearing loss was considered moderate when thresholds fell between 41- and 70-dB HL, whereas thresholds from 71 to 90 dB HL indicated severe hearing loss. Finally, individuals with hearing thresholds exceeding 91 dB HLs were classified as having profound hearing loss.

### Definitions and Measurements

In this study, hearing loss was defined as a hearing threshold greater than 25 dB at one or more frequencies in either ear. Participants with sickle cell disease (SCD) were considered to be in a steady state if they had no crisis symptoms, such as pain or pallor, for a period of four to six weeks, as confirmed through clinical evaluation. The severity of crises was assessed retrospectively using the Wong-Baker FACES Pain Rating Scale, where scores of 0–2 indicated mild crises, 4–6 represented moderate crises, and 8–10 signified severe crises. Socioeconomic status was determined using Oyedeji’s method, which is based on parental occupation and education scores. Nutritional status was evaluated using weight-for-height and body mass index (BMI) in accordance with World Health Organization (WHO) growth standards.

### Data Analysis

Data were analyzed using IBM SPSS Statistics for Windows, Version 20.0. Descriptive statistics (frequencies, means, and standard deviations) were used to summarize the participant characteristics. Categorical variables were compared using the chi-square or Fisher’s exact tests, while continuous variables were compared using independent t-tests. In the SCD cohort, multivariate logistic regression identified predictors of SNHL (e.g., white blood cell count, platelet count, clinic attendance, medication adherence, and crisis frequency). Statistical significance was set at p < 0.05, with results reported as odds ratios (ORs) and 95% confidence intervals (CIs).

### Ethical Approval

Ethical approval for this study was obtained from the Health Research Ethics Committee of Ahmadu Bello University Teaching Hospital, Zaria, Nigeria. Written informed consent was obtained from parents or caregivers prior to participation, along with assent from participants aged ≥7 years.

## Results

*Sociodemographic Characteristics of the Participants*

A total of 125 participants aged 5–16 years were enrolled, comprising 68 males (54.4%) and 57 females (45.6%), yielding a male-to-female ratio of 1.2:1 and a mean age of 10.17 ± 3.35 years. The chi-square test revealed no significant differences in age and sex distribution between cases (SCD participants) and controls (χ² = 0.75; p = 0.69), confirming effective matching. Among the SCD participants, 85 (68%) had the HbSS genotype, 6 (4.8%) had HbSC, and 34 (27.2%) had (HbSS+F). All the controls had the HbAA genotype. The ethnicity distribution included Hausa (43.2%), Yoruba (7.2%), Igbo (1%), and others (7.2%). Most SCD participants (83.3%) belonged to the low socioeconomic class. Poor clinic attendance was reported in 38.4% of SCD cases, whereas 43.2% exhibited poor adherence to routine medications (Table 1).

**Table 1:** Sociodemographic Characteristics of the Participants (Case and Control)

	**SCD (n=125)**	**Controls (n=125)**	**Total (n-250)**	**Statistics**
**Variables**	**n (%)**	**n (%)**	**n (%)**	**χ** ^2^ **p**
Age (in years) 5 – 8 >8 - 12 >12 - 16 Gender Male Female Ethnic Group Hausa Yoruba Igbo Others Hb Phenotypes HbAA HbSS HbSC HbSS+F Social Class Class I Class II Class III Class IV Class V Weight-for-Age Normal weight Overweight Obese Moderate underwt Severe underweight Height-for-Age Normal height Moderate stunting Severe stunting	45 (36.0) 47 (37.6) 33 (26.4) 68 (54.4) 57 (45.6) 106 (84.4) 9 (7.2) 1 (0.8) 9 (7.2) - 85 (68.0) 6 (4.8) 34 (27.2) 6 (4.8) 16 (12.8) 32 (25.6) 52 (41.6) 19 (15.2) 53 (42.4) - - 34 (27.2) 38 (30.4) 80 (64.0) 34 (27.2) 11 (8.8)	45 (36.0) 47 (37.6) 33 (26.4) 68 (54.4) 57 (45.6) 63 (50.4) 17 (13.6) 20 (16.0) 25 (20.0) 125 (100) - - - 11 (8.8) 27 (20.6) 48 (38.4) 31 (24.8) 8 (6.4) 107 (85.6) 4 (3.2) 3 (2.4) 7 (5.6) 4 (3.2) 107 (85.6) 14 (11.2) 4 (3.2)	90 (36.0) 94 (37.6) 66 (26.4) 136 (54.4) 114 (45.6) 169 (67.6) 26 (10.4) 21 (8.4) 34 (13.6) 125 (50.0) 85 (34.0) 6 (2.4) 34 (13.6) 17 (6.8) 43 (17.2) 80 (32.0) 83 (33.2) 27 (10.8) 160 (64.0) 4 (1.6) 3 (1.2) 41 (16.4) 42 (16.8) 187 (74.8) 48 (19.2) 15 (6.0)	38.122 ˂ 0.001* 17.279 ˂ 0.002* Fishers exert 74.365 ˂0.001* 15.498 ˂0.001*

SCD = Sickle Cell Disease; *Statistically Significant; Class I-III = Low Social Class; Class IV & V= High Social Class;

*Mean Middle Ear Volumes in Participants and Controls*

The mean middle ear volumes for the right and left ears in SCD participants were 0.74 ± 0.10 mL and 0.73 ± 0.12 mL, respectively. In controls, the mean volumes were 0.72 ± 0.09 mL (right ear) and 0.71 ± 0.07 mL (left ear). 

Statistical analysis using independent t-tests revealed no significant differences in middle ear volumes between SCD participants and controls for either ear. For the right ear, t = 1.660, p = 0.09; left ear, t = 1.610, p = 0.10 (degrees of freedom = 248). These findings indicate comparable middle ear volumes between the two groups. 

*Prevalence of Sensorineural Hearing Loss Among Participants*

Bilateral sensorineural hearing loss (SNHL) was identified in 32 (25.6%) of the 125 participants with SCD, whereas no hearing loss was observed in the controls. The prevalence of SNHL varied across age groups (Table 2), with the highest rates in the 8–12 and 12–16 years age groups and the lowest in the 5–8 years age group (χ² = 3.453, p = 0.178) (Table 3). A significant male preponderance was observed in 21 (30.9%) males and 11 (19.3%) females (p = 0.015). The mean age of participants with SNHL (11.0 ± 3.2 years) was slightly higher than those without SNHL (9.81 ± 3.3 years), though this difference was not statistically significant (t = 1.751, p = 0.082). The prevalence of SNHL also varied across hemoglobin phenotypes (Table 4). 

**Table 2:** Prevalence of Sensorineural hearing loss in Subjects and Controls

	**SCD (n=125)**	**Control (n=125)**	**Total n=250**
**Variables**	**n (%)**	**n (%)**	**n (%)**
Hearing loss	32 (25.6)	--	32 (12.8)
No hearing loss	93 (74.4)	125 (100)	218 (87.2)
Total	125 (100)	125 (100)	250 (100)

**Table 3:** Prevalence of SNHL among Subjects with SCD across the Age groups

	**Sickle Cell Disease - Age Groups (Years)**			
	**5- 8 yrs (n=45)**	**>8 -12 yrs (n=47)**	**>12-16 yrs (n=38)**	**Total(n=125)**
**Variables**	**n (%)**	**n (%)**	**n (%)**	**n (%)**
Hearing loss	8 (17.8)	12 (25.5)	12 (36.4)	32 (25.6)
No hearing loss	37 (82.2)	35 (74.5)	21 (63.6)	93 (74.4)
Total	45 (100)	47 (100)	33 (100)	125 (100)

*SCD = Sickle cell disease; yrs = years*

**Table 4:** Prevalence of SNHL among haemoglobin phenotypes in the Subjects

	**Haemoglobin phenotypes**			
	**HbSS (n=85)**	**HbSC (n=6)**	**HbSS+F (n=34)**	**Total(n=125)**
**Variables**	**n (%)**	**n (%)**	**n (%)**	**n (%)**
Hearing loss	28 (32.9)	3 (50.0)	1 (2.9)	32 (25.6)
No hearing loss	57 (87.1)	3 (50.0)	33 (97.1)	93 (74.4)
Total	85 (100)	6 (100)	34 (100)	125 (100)

*SNHL= Sensorineural hearing loss; SCD= Sickle cell disease; HbSS = Sickle cell anaemia; HbSS+F = Sickle Cell Anaemia with persistent fetal haemoglobin; HbSC = SC disease.*

*Logistic Regression Analysis of Risk Factors for SNHL Among Subjects with Sickle Cell Disease*

Binary logistic regression analysis identified multiple significant risk factors for sensorineural hearing loss (SNHL) in children with sickle cell disease (SCD). Early onset of the first sickle cell crisis (<5 years) was strongly associated with SNHL (adjusted OR, 1.163; 95% CI, 1.071–1.262; p < 0.001). Participants with SNHL had a significantly lower mean age at diagnosis and first crisis (1.09 ± 0.3 years) compared to those without SNHL (1.72 ± 0.8 years, p < 0.001). Homozygous sickle hemoglobin (HbSS) was associated with a higher risk of SNHL than HbSC or (HbSS+F) genotypes (adjusted OR: 5.056, 95% CI: 1.640–15.583, p = 0.002) (Table 5). Participants experiencing combined vaso-occlusive and anemic crises had a significantly higher risk of SNHL than those experiencing isolated vaso-occlusive crises (adjusted OR, 7.462; 95% CI, 3.067–18.181; p < 0.001). Frequent severe crises (>3/year) (adjusted OR, 23.250; 95% CI, 8.914–60.642), >3 hospitalizations/year (adjusted OR, 23.250; 95% CI, 8.914–60.642), and frequent blood transfusions (≥2/year) (adjusted OR, 52.632; 95% CI, 16.390–166.667) were strongly associated with SNHL (Table 5).

**Table 5:** Binary Logistic Regression Analysis of Risk factors for Sensorineural Hearing loss Among Subjects with SCD

	**Sickle Cell Disease (n = 125)**					**Binary Logistic Regression Analysis**			
	**SCD w/o HL**	**SCD with HL**	**Total**	**Statistics**			**95% C.I. for Odds Ratio**		
**Variables**	**(n = 93)**	**(n = 32)**	**(n =125)**	**Chi-square**	**P - value**	**OR**	**Lower**	**Upper**	
Nutritional Status ˂ - 2SD Z-score ≥ - 2SD to ˂ + 1SD Haemoglobin types Hb SS genotype Others (SS+F& SC) Socioeconomic class Low (Class IV-V) High (Class I-III) Age at Diagnosis of SCD Less than 5 yrs Greater than 5 yrs Age at 1st onset of crisis Less than 5 yrs Greater than 5 yrs Type Of SCD Crisis VOC + Anaemia VOC Only	61 (65.6) 32 (34.4) 54 (58.1) 39 (41.9) 48 (51.2) 45 (48.8) 80 (86.0) 13 (14.0) 80 (86.0) 13 (14.0) 19 (20.4) 74 (79.6)	23 (71.9) 9 (28.1) 28 (87.5) 4 (12.5) 23 (71.9) 9 (28.1) 32 (100) - 32 (100) - 21 (65.5) 11 (34.4)	84 (67.2) 41 (32.8) 82 (65.5) 43 (34.4) 71 (56.8) 54 (43.2) 112(89.6) 13 (10.4) 112(89.6) 13(10.4) 40 (32.0) 85 (68.0)	0.426 9.141 3.983 Fishers 4.992 Fishers 4.992 22.348	0.541 0.002* 0.046* exert 0.025* exert 0.025* ˂ 0.001*	1.341 5.056 2.398 1.163 1.163 7.462	0.555 1.640 1.002 1.071 1.071 3.067	3.236 15.583 5.714 1.262 1.262 18.181	

*Statistically significant differences; SCD= Sickle cell disease; C.I. = Confidence interval; HL = Hearing Loss; OR=Odds Ratio; VOC=Vaso-occlusive *crisis*

*Socioeconomic and Nutritional Factors*

Lower socioeconomic status (classes IV and V) was associated with a higher risk of SNHL compared to higher socioeconomic status (adjusted OR, 2.398; 95% CI, 1.002–5.714; p = 0.046). While nutritional status did not significantly differ between the groups (p = 0.541), well-nourished participants (≥-2SD to <+1SD) showed a trend toward lower SNHL risk compared to undernourished participants (adjusted OR, 1.341; 95% CI, 0.555–3.236) (Tables 5 and 6).

**Table 6:** Binary Logistic Regression Analysis of Risk factors for Sensorineural Hearing loss Among Subjects with SCD (Cont’d)

	**Sickle Cell Disease (n = 125)**					**Binary Logistic Regression Analysis**		
	**SCD w/o HL**	**SCD with HL**	**Total**	**Statistics**			**95% C.I. for Odds Ratio**	
**Variables**	**(n = 93)**	**(n = 32)**	**(n = 125)**	**Chi-square**	**P - value**	**Odds ratio**	**Lower**	**Upper**
Severity Of Crisis Severe crisis Mild-Moderate crisis Frequency of SCD crisis ≥ 3 episodes of crisis/yr ˂ 3 episodes of crisis/yr No of admissions/year ≥ 3 admissions per year ˂ 3 admissions per year Blood Transfusion ≥ 1 unit of blood/year None/occasional Clinic Attendance Miss ≥ 2 clinics/year Regular clinic attendance Routine Medications Miss ≥ 3 doses/month Regular on medications	14 (15.1) 79 (84.9) 4 (04.3) 89 (95.7) 4 (04.3) 89 (95.7) 6 (07.5) 86 (92.5) 18 (19.4) 75 (80.6) 25 (26.9) 68 (73.1)	24 (75.0) 8 (25.0) 32 (100) 00 (0.0) 32 (100) 00 (0.0) 26 (81.2) 6 (18.8) 30 (93.8) 2 (06.2) 29 (90.6) 3 (09.4)	38 (30.4) 87 (69.6) 36 (28.8) 89 (71.2) 36 (28.8) 89 (71.2) 33 (26.4) 92 (73.6) 48 (38.4) 77 (61.6) 54 (43.2) 71 (56.8)	40.436 106.332 106.332 66.596 55.706 39.424	˂ 0.001* ˂ 0.001* ˂ 0.001* ˂ 0.001* ˂ 0.001* ˂ 0.001*	16.946 23.250 23.250 52.632 62.500 26.320	6.329 8.914 8.914 16.390 13.704 7.350	45.454 60.642 60.642 166.667 333.330 90.910

*Statistically significant differences; SCD = Sickle cell disease; C.I. = Confidence interval; HL = Hearing

*Hematological Parameters*

Significant differences in hematological parameters were observed between SCD participants with and without SNHL. Those with SNHL had lower mean hematocrit levels (22.5 ± 1.7 g/dL vs. 24.7 ± 2.8 g/dL, p = 0.032), higher mean white blood cell counts (19.5 ± 4.4 × 10⁹/L vs. 13.2 ± 5.3 × 10⁹/L, p < 0.001), and higher mean platelet counts (478.0 ± 76.3 × 10⁹/L vs. 308.4 ± 78.0 × 10⁹/L, p < 0.001) (Table 6).

*Compliance and Health Utilization*

Noncompliance with clinic attendance and medications significantly predicted SNHL. Participants with >2 clinic visits per year had markedly higher odds of SNHL (adjusted OR: 28.668, 95% CI: 4.879–168.458), as did those missing >3 medication days per month (adjusted OR: 9.634, 95% CI: 1.830–50.718) (Table 6).

*Multivariate Regression Analysis of Predictors for Sensorineural Hearing Loss in SCD*

Multivariate logistic regression analysis identified key independent predictors of sensorineural hearing loss (SNHL) in children with sickle cell disease (SCD). Poor clinic attendance, defined as missing more than two clinic visits per year, was strongly associated with an increased risk of SNHL. Similarly, non-adherence to routine medications, defined as missing >3 days of prescribed medication per month, significantly predicted SNHL. Clinical factors such as severe sickle cell crises requiring hospitalization have also emerged as significant predictors. Hematological parameters, including elevated white blood cell counts (>13.5 × 10⁹/L) and platelet counts (>450 × 10⁹/L), were independently associated with SNHL (Table 7).

**Table 7:** Multivariate logistic regression of clinical variables on SNHL

**Multivariate Regression Model**	**Statistics**		**95% CI for OR**	
	**P- value**	**Odds Ratio**	**Lower**	**Upper**
**Categorical Variables**				
Poor clinic attendance (≥ 3times/year) Non-adherence to routine medications Severe form of sickle cell crisis Type of sickle cell crisis Sickle cell disease (Hb SS) Nutritional status (Malnourished) Socioeconomic class (lower social class) Age at onset of crisis (Age ≤ 5 years)	˂ 0.001* 0.008* 0.011* 0.703 0.903 0.560 0.667 0.691	28.668 9.634 0.106 0.703 1.111 1.591 1.390 0.695	4.879 1.830 0.019 0.156 0.205 0.334 0.310 0.000	168.458 50.718 0.598 3.500 6.019 7.575 6.228 0.000
**Continuous Variables**				
High platelet counts > 450 x 10^9^/l High WBC counts > 13.5 x 10^9^/l	˂ 0.001* 0.002*	1.035 1.070	1.020 1.070	1.050 1.365

*Statistically significant differences; CI = Confidence interval; OR= *Odds ratio; WBC=* *White Blood Cell*

## Discussion

The prevalence of sensorineural hearing loss (SNHL) in this study was 25.6% among children and adolescents with sickle cell disease (SCD), with no cases observed in the HbAA control group. 

This finding underscores SNHL as a significant complication of SCD, which is consistent with prior studies (4,5,37-42). The exclusive bilateral presentation of SNHL in SCD participants aligns with the hypothesized pathophysiology of cochlear vulnerability to ischemic damage due to vaso-occlusive events in terminal vessels, such as the labyrinthine artery. A significant association was observed between hemoglobin phenotypes and SNHL risk. Participants with HbSS had fivefold higher odds of SNHL than those with HbSC or (HbSS+F). The lower incidence of SNHL in HbSS+F group may reflect the protective role of fetal hemoglobin (HbF), which mitigates SCD severity (13,43,44). This contrasts with the studies by Friedman et al. (15) and Crawford et al. (45), who reported higher SNHL rates in HbSC patients. These discrepancies likely stem from differences in cohort composition, as our study had a higher proportion of HbSS participants, whereas prior studies included larger HbSC subgroups.

Males exhibited a higher SNHL prevalence (30.9%) than females (19.3%), consistent with the findings of Aderibigbe et al. in Nigeria (46). However, this conflicts with Al-Okbi et al.’s study in Oman, where females had more severe hearing loss (8). Variations in menstrual blood loss, access to healthcare, and study methodologies may have contributed to these differences. Notably, only 0.2% of adolescent females in our cohort attained menarche, limiting the applicability of hypotheses linking lower packed-cell volumes in females to cochlear damage (18,46–50).

Early onset of crises (<5 years), frequent hospitalizations (≥3/year), and blood transfusions (≥2/year) were strongly associated with SNHL. These factors reflect disease severity because chronic hemolysis and vaso-occlusion likely impair cochlear oxygenation, leading to ischemic damage (16,20,51). This aligns with studies linking severe SCD phenotypes to higher SNHL risks (4,8,9).

SNHL was more prevalent among participants from low socioeconomic backgrounds (71.9%) and those with malnutrition (62.4% underweight and 40.6% stunted). Lower socioeconomic status and malnutrition were associated with a two-fold higher risk of SNHL, consistent with prior research (7, 46). Poverty and limited education are likely to exacerbate barriers to care, delay treatment, and increase complication rates. Malnutrition, which is common in SCD owing to metabolic demands and poor dietary intake, may further potentiate disease severity (2,52).

This study identified non-compliance with clinic appointments and medication non-adherence as significant predictors of sensorineural hearing loss (SNHL) in children and adolescents with sickle cell disease (SCD). Participants missing ≥6 clinic visits annually or non-adherent to medications for ≥5 days/month were 52 and 26 times more likely to develop SNHL, respectively, than those with regular follow-up and adherence. These findings align with those of Lori et al. (53) and Shannon et al. (54), who highlighted barriers such as poverty, geographic distance, transportation challenges, out-of-pocket costs, and limited access to specialized care as drivers of poor adherence in chronic diseases. Structured SCD clinics offering specialized care, education, and early intervention are critical for reducing complications, such as SNHL, in resource-limited settings.

This study reinforces the role of hematological markers in the risk of SCD in patients with SCD. Elevated white blood cell (WBC) counts (≥13.5 × 10⁹/L) and platelet counts (≥450 × 10⁹/L) were strongly associated with SNHL, consistent with Seo et al. (55) and Kum et al. (56). High WBC counts, linked to vaso-occlusive crises and platelet-driven hypercoagulability, likely contribute to cochlear ischemia. Low hematocrit levels (≤22.5 g/dL) also correlated with SNHL, supporting the findings of Grant et al. (57) on anemia and auditory nerve dysfunction. Although Aderibigbe et al. (46) proposed menstrual blood loss as a risk factor in females, this was less relevant in our cohort due to the low menarche rate (0.2%). In contrast, Todd et al. (21) in Jamaica highlighted population-specific variations in the pathophysiology of SNHL.

Multivariate analysis revealed four independent predictors of sensorineural hearing loss (SNHL) in individuals with sickle cell disease (SCD). Poor clinic attendance, defined as missing two or more visits annually, increases the risk of SNHL by delaying interventions that could mitigate disease progression. Medication non-adherence, characterized by missing three or more prescribed doses per month, further heightened the risk of SNHL by reducing the efficacy of disease management. Severe vaso-occlusive crises requiring hospitalization directly contribute to SNHL through ischemic damage to the cochlea, likely exacerbated by acute blood flow disruptions. Additionally, elevated white blood cell (WBC) and platelet counts have emerged as critical markers, reflecting heightened inflammation and thrombosis that worsen microvascular injury, a hallmark of SCD pathophysiology. These findings underscore the interplay among clinical management, disease severity, and systemic inflammation in driving auditory complications in this population.

These findings underscore the importance of adherence to care, aggressive crisis management, and monitoring of hematological parameters to mitigate the risk of SNHL in SCD.

## Conclusion

This study underscores the high prevalence of sensorineural hearing loss (SNHL) among children and adolescents with sickle cell disease (SCD), highlighting it as a significant complication. Key findings revealed significant associations between elevated white blood cells and platelet counts, low hematocrit levels, and SNHL, suggesting that disease severity plays a critical role in hearing loss pathogenesis. Poor clinic attendance, medication non-adherence, low socioeconomic status, and malnutrition were identified as modifiable predictors of SNHL, whereas higher fetal hemoglobin (HbF) levels appeared to be protective. Gender differences were also observed, with males exhibiting a higher prevalence of SNHL. These findings emphasize the complex interplay of biological, environmental, and healthcare-related factors in the development of SNHL among individuals with SCD.

### Limitation of the Study

This study was conducted in a single-center setting, which may limit the generalizability of the findings to a broader population. Additionally, the cross-sectional design restricts the ability to establish adequate causal relationships between identified risk factors and sensorineural hearing loss (SNHL). Self-reporting of socioeconomic status, clinical severity of crises, and other variables introduces potential recall and reporting bias, which could affect the accuracy of the data.

### Recommendations

To address the burden of SNHL among children and adolescents with sickle cell disease (SCD), routine audiological screening should be integrated into standard care to facilitate early detection and management. Specialized SCD clinics with multidisciplinary care should be established, particularly in resource-limited settings, to improve access to comprehensive care. Targeted health education programs are essential to enhance medication adherence and clinic attendance, whereas nutritional support initiatives should address the high prevalence of malnutrition among this population. 

Furthermore, policies aimed at reducing socioeconomic barriers to health care, such as subsidized treatments and expanded health insurance coverage, should be prioritized. Finally, longitudinal research is needed to explore the causal relationships between risk factors and SNHL as well as to evaluate the effectiveness of preventive and therapeutic strategies.
